# A-to-I nonsynonymous RNA editing was significantly enriched in the ubiquitination site and correlated with clinical features and immune response

**DOI:** 10.1038/s41598-022-18926-x

**Published:** 2022-09-05

**Authors:** Haixia Li, Jianjun Wang, Juchuanli Tu

**Affiliations:** 1 Department of Obstetrics and Gynecology, Beijing Tiantan Hospital, Capital Medical University, Bejing, China; 2 CAS Key Laboratory of Microbial Physiological and Metabolic Engineering, Institute of Microbiology, Chinese Academy of Sciences, Beijing, China; 3 Fudan University Shanghai Cancer Center and Institutes of Biomedical Sciences; Cancer Institutes; Key Laboratory of Breast Cancer in Shanghai; The Shanghai Key Laboratory of Medical Epigenetics; The International Co-Laboratory of Medical Epigenetics and Metabolism, Ministry of Science and Technology; Shanghai Medical College, Fudan University, Shanghai, 200032 China

**Keywords:** Cancer, Computational biology and bioinformatics, Genetics

## Abstract

RNA editing is a post-transcriptional process that alters RNA sequence in a site-specific manner. A-to-I editing is the most abundant as well as the most well-studied type of RNA editing. About 0.5% of A-to-I editing sites were located in the coding regions. Despite of thousands of identified A-to-I nonsynonymous editing sites, the function of nonsynonymous editing was poorly studied. Here, we found that the nonsynonymous editing was significantly enriched in the ubiquitination site, compared to the synonymous editing. This enrichment was also in a modification type dependent manner, since it was not significantly enriched in other modification types. This observation was consistent with previous study that the codons for lysine (AAG and AAA) were enriched in the preferred deamination site for RNA editing. The peptides from proteomic data in CPTAC supported that mRNAs harboring edited ubiquitination sites can be translated into protein in cells. We identified the editing sites on ubiquitination site were significantly differential edited between tumor and para-tumor samples as well as among different subtypes in TCGA datasets and also correlated with clinical outcome, especially for the nonsynonymous editing sites on GSTM5, WDR1, SSR4 and PSMC4. Finally, the enrichment analysis revealed that the function of these above genes was specifically enriched in the immune response pathway. Our study shed a light on understanding the functions of nonsynonymous editing in tumorigenesis and provided nonsynonymous editing targets for potential cancer diagnosis and therapy.

## Introduction

RNA editing is one of the post-transcriptional processes which alter RNA sequence in a site-specific manner^1^. RNA editing was first discovered by Benne et al. and they found that the mitochondrial pre-mRNAs in trypanosomes could be post-transcriptionally edited by the insertion or deletion of uridylate (U) residues^2^. With the deepening of research, people have discovered that RNA editing is a widespread process and has been observed to occur on mRNAs, tRNAs, rRNAs and RNAs in nucleus, mitochondrial and chloroplast^1^.

To date, there are more than one hundred types of RNA editing which have been identified^3^. The two of the most widespread types of RNA editing are adenosine-to-inosine (A-to-I) editing and cytosine-to-uracil (C-to-U) editing in mammals^4^. Among these two types of RNA editing, A-to-I editing is the most abundant as well as the most well-studied RNA editing type^4,5^. In mammals, ADAR genes, ADAR, ADARB1 and ADARB2, are responsible for the A-to-I editing^6,7^. ADARB2 are uniquely expressed in specific tissues, ADAR and ADARB1 is widely expressed in various tissues and responsible for the majority of editing activity^3^. It is well known that most RNA editing in human occurs in the Alu sequences^5^. AG-rich motif was witnessed in the local sequence for A-to-I editing site^8,9^.

Taking the advantage of high-throughput sequencing methods, tens of thousands of A-to-I editing sites were identified in several species including human, mouse and rat^10–12^. Among these A-to-I editing sites, around 50% of editing sites were located on 3’UTR of genes and only about 0.5% of A-to-I editing sites were located in the coding regions^13,14^. Several hypothesizes were proposed to explain the function of nonsynonymous editing, such as resulting in the alteration of splicing pattern and contributing to the proteomic diversity^15,16^.

The A-to-I RNA editing is also reported to be involved in tumorigenesis by mediating several important biological processes^17–19^. Hundreds of A-to-I RNA editing sites were identified as differential editing sites and correlated with clinical outcome in cancer^13^. Besides, several previous studies showed that alteration of amino acid due to RNA editing could create novel antigen site (neo-antigens) and may contribute to the immune therapy in cancer^4,20,21^. However, only a small number of A-to-I nonsynonymous editing were uncovered to function in cells, despite of hundreds of discovered nonsynonymous editing events^22–26^. The function of most of A-to-I nonsynonymous editing sites was largely unknown.

Here, we reported that the nonsynonymous A-to-I editing sites were significantly enriched in the ubiquitination sites. This enrichment was specific, compared to the other types of modification including phosphorylation, acetylation and etc. We also confirmed this significant and specific enrichment in The Cancer Genome Atlas (TCGA) datasets. By analyzing the proteome data in CPTAC datasets, we identified peptides harboring the ubiquitination sites altered by RNA editing. This observation supported that these nonsynonymous editing sites can be translated into peptide instead of degradation before translation. Furthermore, the editing level of some editing sites on ubiquitination site was significantly different between tumor and para-tumor as well as among different subtypes. Survival analysis revealed these sites were also significantly correlated with clinical outcome, indicating its potential functions in cancer. The GSEA enrichment analysis show that the genes exposed to the nonsynonymous editing on the ubiquitination sites are significantly and uniquely enriched in the functions related to the immune response pathway.

Our analysis revealed a possible novel function of nonsynonymous A-to-I RNA editing to regulate ubiquitination and shed a light on understanding its functions in tumorigenesis.

## Results

### Nonsynonymous A-to-I RNA editing was significantly and specifically enriched in the ubiquitination sites

In order to explore the potential function of nonsynonymous A-to-I RNA editing in the human genome, we downloaded the A-to-I RNA editing sites from DARNED and REDIportal databases^11,12^. We also incorporated an A-to-I RNA editing dataset from a recent study and named it as “Gabay2022”^8^. The aim of these three databases is to establish comprehensive records of A-to-I RNA editing events independent of tissue, disease and patients. Especially for REDIportal, it recorded over 15 million of A-to-I RNA editing events derived from 9642 human RNA-seq samples from 549 individuals (31 tissues and 54 body sites) of the GTEx project which help us to investigate the potential function of nonsynonymous A-to-I RNA editing in an unbiased manner.

**Figure 1:**
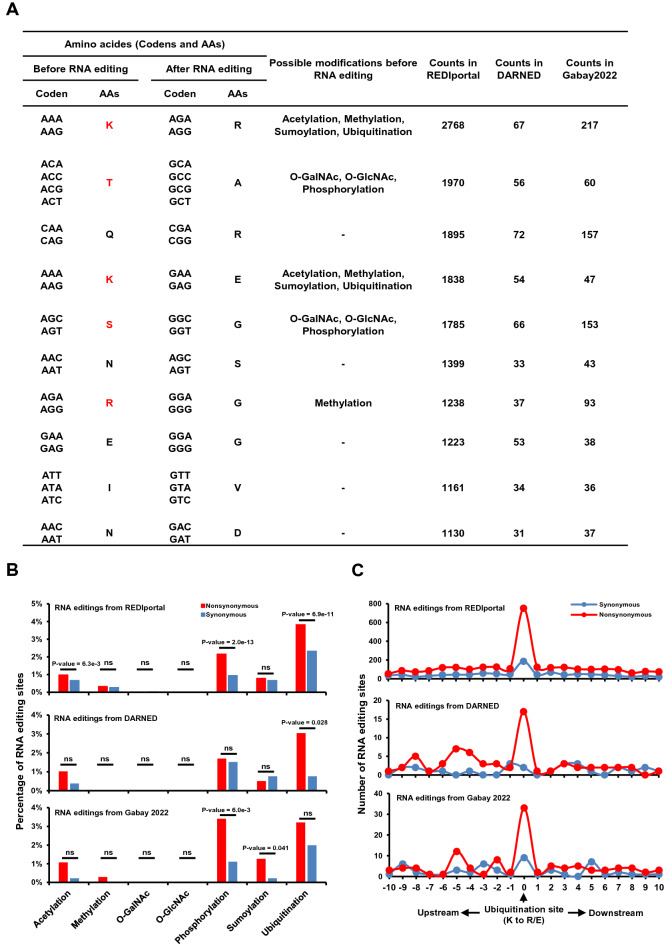
Overlaps between RNA editings and protein modification sites. (**A**) Table summarizing the frequency of amino acids (AAs) affected by nonsynonymous RNA editing in three RNA editing databases. (**B**) Bar plot demonstrating the percentage of nonsynonymous RNA editing in three RNA editing databases located in the protein modification sites. The P-value was computed by one-sided “fisher.test” function in R. (**C**) The distribution of synonymous (blue) and nonsynonymous (red) RNA editing from three RNA editing databases on ubiquitination site and flanking regions. (**D**) Venn plotting showed the overlaps between ubiquitination and acetylation sites in PhosphoSitePlus database. (**E**) Bar plot demonstrating the percentage of nonsynonymous RNA editing in REDIportal database located in the ubiquitination unique and acetylation unique sites. The P-value was computed by one-sided “fisher.test” function in R. (**F**) The distribution of synonymous (blue) and nonsynonymous (red) RNA editing from REDIportal database on ubiquitination unique site and flanking regions (upper) and on acetylation unique site and flanking regions (bottom).

From here on, we called A-to-I RNA editing as RNA editing for short. Firstly, we summarized the counts of changes of amino acids (AAs) due to the nonsynonymous RNA editing in three databases and sorted it in descendent order. The results showed that the several AAs frequently affected by nonsynonymous RNA editing in all three databases were the potential target sites for protein modification (Fig. 1A; Suppl. Tables 1, 2 and 3). Furthermore, the AAs edited by RNA editing usually converted into the AAs which got very low possibility to be modified as the original AAs (Suppl. Fig. S1A). For example, almost of all ubiquitination happened on Lysine. On the contrary, almost of none ubiquitination happened on arginine, which was converted from lysine due to RNA editing (Fig. 1A; Suppl. Fig. S1A). It was worth noting that the frequency of conversion from lysine to arginine (K to R) due to RNA editing in all three databases was the highest among these AAs which were the potential sites for protein modification (Fig. 1A; Suppl. Fig. S1A). Furthermore, previous studies also supported us observations and showed that the codons for lysine (AAG and AAA) were enriched in the preferred deamination site for RNA editing^8,9^.

The above observations lead us to make the hypothesis that nonsynonymous RNA editing may alter the AA code of modification and thus regulate the modification level. This hypothesis inspired us to investigate the overlaps between the nonsynonymous RNA editing and protein modification sites. We downloaded common modification types from PhosphoSitePlus^27^. The PhosphoSitePlus collected modification sites from near 10,000 previous studies, some of which were high-throughput MS data (Suppl. Fig. S1A). The huge amount of data guaranteed the comprehensive coverage of modification sites in the human genome and thus show no tissue, patients and disease specific. Indeed, we witnessed that varied percentage of nonsynonymous RNA editing sites overlapped with different protein modification types and the overlaps of nonsynonymous RNA editing sites from three databases showed similar pattern (Fig. 1B). For example, none of nonsynonymous RNA editing was located on the O-GalNAc and O-GlcNAc sites. On the contrary, about 3% of RNA editing sites from all three databases were located on the ubiquitination sites which was the highest overlapping percentage.

To test the significant overlaps between nonsynonymous RNA editing and protein modification sites, we separately summarized the percentage of nonsynonymous and synonymous RNA editing sites located on the modification sites and then compared these two percentages. Since both of the synonymous and nonsynonymous RNA editings were located on the coding region and the only difference is whether it altered the coding of amino acids or not, synonymous RNA editing was a perfect control to test the specificity and significance of enrichment for nonsynonymous RNA editings. The significant test showed that the difference between nonsynonymous and synonymous RNA editing located on ubiquitination site was significant in REDIportal and DARNED database (Fig. 1B). It showed the same pattern in Gabay2022 and was not significant. Besides, the difference between nonsynonymous and synonymous RNA editing overlapped with phosphorylation was also significant in REDIportal and Gabay2022.

We also plotted the distribution of nonsynonymous RNA editing sites on the protein modification sites and the flanking regions. The results showed that nonsynonymous RNA editing sites from all three databases were enriched in the ubiquitination sites, compared to the flanking regions (Fig. 1C). Furthermore, the synonymous RNA editing sites from all three databases showed no such significant enrichment (Fig. 1C). It was consistent with the observation of significant overlaps between nonsynonymous RNA editing and ubiquitination site. For the other modifications, sumoylation and acetylation showed significant enrichment in REDIportal and Gabay2022 (Suppl. Fig. S2A, S2B and S2C). Besides, phosphorylation and methylation showed a relative weak enrichment only in REDIportal (Suppl. Fig. S2A).

### The enrichment of nonsynonymous RNA editing in the ubiquitination site is not due to the codon tendency

Previous studies showed that the codons for lysine (AAG and AAA) were enriched in the motif for RNA editing^8,9^. Besides, we also noticed that the codons for lysine have two adenosines which may be the potential targets for nonsynonymous editing and the enrichment may just be codon dependent and K has more likely to be edited than any other amino acids. Thus, it reflected specific enrichment for codon instead of ubiquitination.

**Figure 2:**
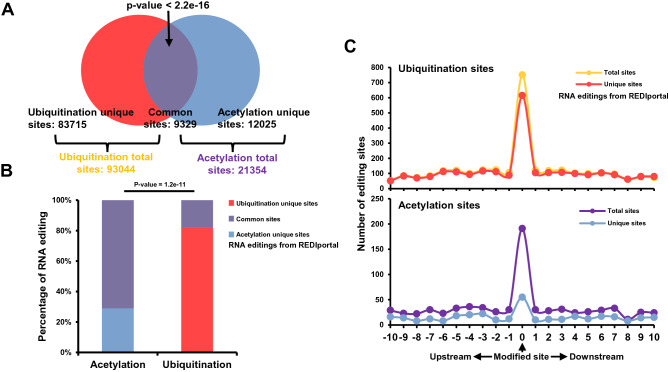
Overlaps between nonsynonymous RNA editings and ubiquitination sites. (**A**) Venn plotting showed the overlaps between ubiquitination and acetylation sites in PhosphoSitePlus database. (**B**) Bar plot demonstrating the percentage of nonsynonymous RNA editing in REDIportal database located in the ubiquitination unique and acetylation unique sites. The P-value was computed by one-sided “fisher.test” function in R. (**C**) The distribution of nonsynonymous RNA editing from REDIportal database on total and ubiquitination unique site and flanking regions (upper) and on total and acetylation unique site and flanking regions (bottom).

To rule out this possibility, we focused on the acetylation, since almost of all acetylation and ubiquitination occurred on K and the number of modification sites for acetylation and ubiquitination were roughly proximal (Suppl. Fig. S1A). The enrichment of nonsynonymous editing from REDIportal on acetylation was significant (Fig. 1B; Suppl. Fig. S2A). Furthermore, these modification sites for acetylation and ubiquitination were significant overlapped (Fig. 2A). If above explanation is right, we could also witness comparable enrichment of nonsynonymous RNA editing from REDIportal database among acetylation unique and ubiquitination unique sites. However, only 30% nonsynonymous editings on acetylation site were from acetylation unique sites. On the contrary, more than 80% nonsynonymous editings on ubiquitination sites were from ubiquitination unique sites (Fig. 2B). The difference was significant. We also witnessed similar results in other two databases (Suppl. Fig. S3A and S3B). Furthermore, the enrichment plot was consistent with above observations (Fig. 2C; Suppl. Fig. S3C and S3D). The significant enrichment of nonsynonymous RNA editing on acetylation site was largely due to the significant overlaps between ubiquitination and acetylation sites on the genome. More importantly, the enrichment on the ubiquitination sites can`t be explained by codon tendency and it reflected specificity for ubiquitination.

In summary, these observations together indicated that nonsynonymous RNA editing sites were significantly and uniquely enriched in ubiquitination sites. The nonsynonymous RNA editing may play a vital role in regulation of the ubiquitination level.

### The nonsynonymous RNA editing was enriched in the ubiquitination sites in cancer

Previous studies identified hundreds of RNA editing sites in various cancer types and found that some of these sites got differential editing level between tumor and para-tumor and were correlated with clinical outcome^13^. Moreover, several previous studies reported that nonsynonymous editing played a vital role in tumorigenesis in different cancer types^22–26^. We were interested in the function of nonsynonymous RNA editing sites located in the ubiquitination sites and explored the potential role of these RNA editing sites during tumorigenesis. In order to achieve this goal, we analyzed the nonsynonymous RNA editing in the TCGA datasets. We defined that RNA editing sites identified in at least two cancer types as common RNA editing sites to ensure the quality of RNA editing sites and used these common RNA editing sites in our following analysis, referring as RNA editing sites in the following analysis in cancer.

**Figure 3:**
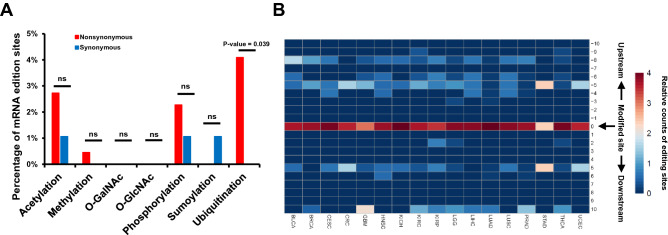
Overlaps between RNA editing and protein modification sites in cancers. (**A**) Bar plot demonstrating the percentage of nonsynonymous RNA editing located in the protein modification sites in TCGA database. The P-value was computed by one-sided “fisher.test” function in R. (**B**) Heatmap displaying the distribution of nonsynonymous RNA editing on ubiquitination site and flanking regions in each cancer type from TCGA database.

In TCGA datasets there were 220 nonsynonymous and 94 synonymous RNA editing sites identified from 17 cancer types in TCGA datasets (Suppl. Fig. S4A and Table 4)^13^. Among these nonsynonymous RNA editing sites, there are about 2–4% of nonsynonymous RNA editing sites located on the protein modification sites (Fig. 3A; Suppl. Fig. S4B). Significant higher percentage of nonsynonymous RNA editing sites were witnessed to be located in the ubiquitination sites and no significance was observed in other modification types when comparing to the synonymous RNA editing sites (Fig. 3A). It was consistent with the observations from three RNA editing database (Fig. 1B). Furthermore, we also investigated the distribution of nonsynonymous RNA editing in ubiquitination sites and flanking regions. The results showed significant enrichment in the modification sites comparing to the flanking region as well as synonymous RNA editing in most of TCGA datasets (Fig. 3B; Suppl. Fig. S4C). The other modification types showed no significant enrichment in most of TCGA datasets (Suppl. Fig. S4D and S4E).

These results indicated that nonsynonymous RNA editing sites identified from various tumor types were also specifically enriched in the ubiquitination sites, but not the other modification types, showing a modification type dependent manner.

### Ubiquitination sites altered by RNA editing were validated on protein level in CPTAC proteomic data

We found that RNA editing was enriched in ubiquitination sites, it was still unknown whether those mRNAs harboring edited ubiquitination sites can be translated into peptides. Although previous studies showed that mRNAs altered nonsynonymous RNA editing could translated into protein to contribute to the proteomic diversity or create neoantigens in cancer^15,20^, it still can`t exclude the possibility that nonsynonymous RNA editing on ubiquitination site was a special exception. If these edited mRNAs can’t be translated, the RNA editing on ubiquitination site could be unlikely to play an important role in cell.

**Figure 4:**
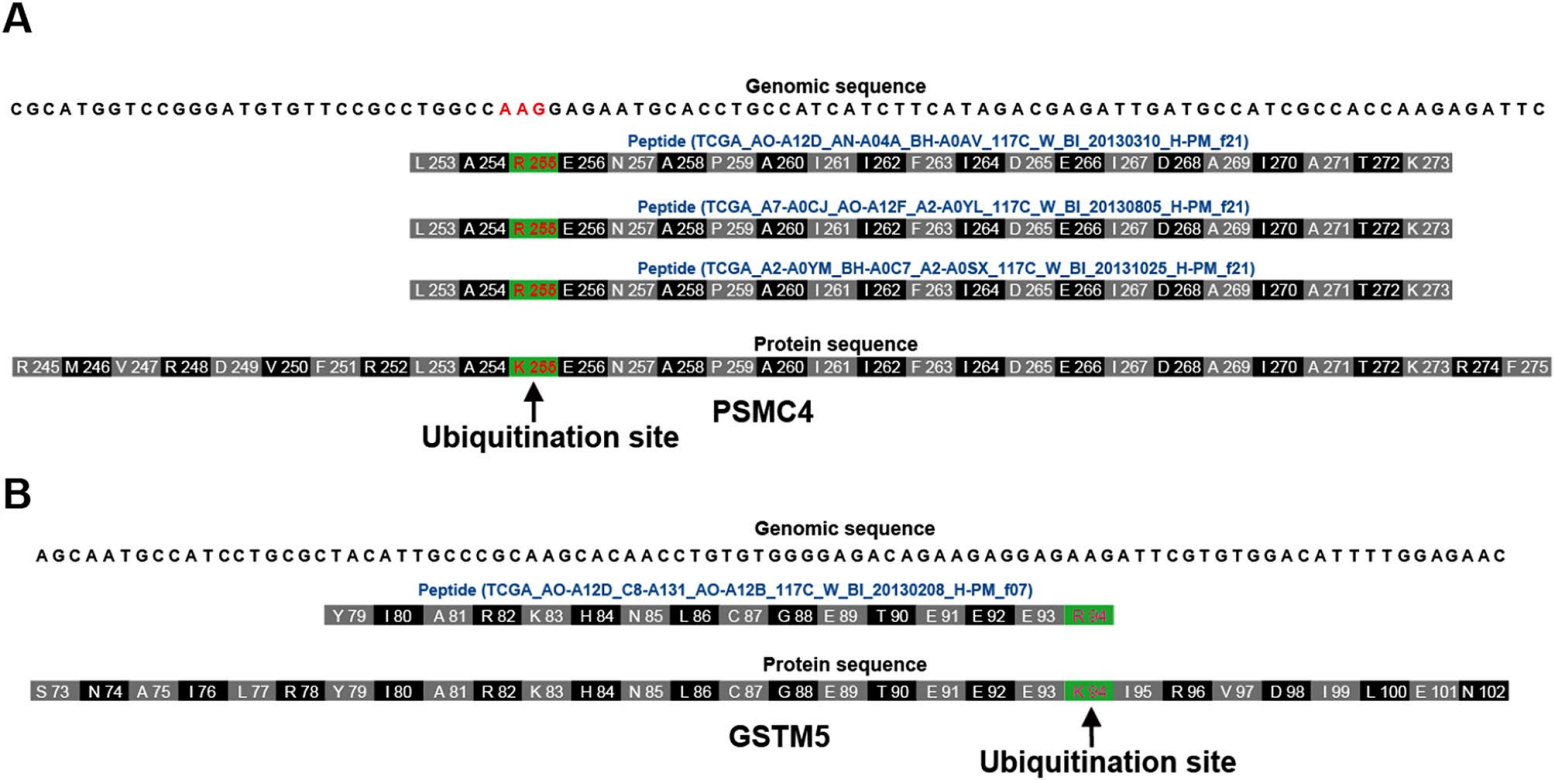
The edited ubiquitination sites by nonsynonymous RNA editing were identified from CPTAC database. (**A**, **B**) Peptides and its genomic location on the human genome. The genome view was snapshotted from UCSC genome browser with slightly modification. The ubiquitination site was highlighted in red and green background. The peptides sequences harboring AAs altered by nonsynonymous RNA editing were highlighted in blue. The altered AAs were also highlighted in red and green background. Genomic sequence was located on the top of each view.

To answer this question, we investigated the proteomic data of breast (BRCA) and ovarian (OV) cancer in clinical proteomic tumor analysis consortium (CPTAC) proteomic data. Indeed, we identified four peptides harboring mismatches on ubiquitination sites from two genes in BRCA and five peptides from four genes in OV (Suppl. Fig. S5A). For example, we identified three peptides harboring the mismatched ubiquitination sites, K255, on PSMC4 and one peptide harboring the mismatched ubiquitination sites, K94, on GSTM5 in BRCA dataset (Fig. 4A,B). In order to filter out the possibility that these mismatched sites were due to somatic mutation not RNA editing, we manually checked the somatic mutations for the patients from which identified mismatched peptides. The results showed that these samples didn`t harbor somatic mutations on these sites, suggesting that these mismatched ubiquitination sites were due to the RNA editing not derived from the somatic mutations.

In summary, we provided the evidence to support that these edited mRNAs can be translated into protein in cell, although not all RNA editing events located on ubiquitination site were identified on peptide level probably due to the depth of proteomic data and the abundance of edited sites.

### The nonsynonymous RNA editing level on ubiquitination site were correlated with clinical features

**Figure 5:**
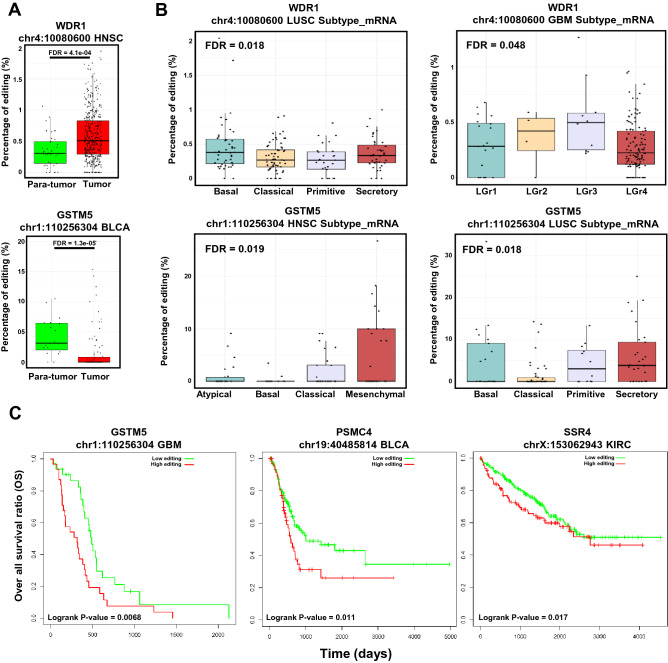
The distribution of nonsynonymous RNA editing on ubiquitination site in TCGA database. (**A**) The boxplot demonstrating the editing level between tumor and para-tumor samples in TCGA dataset. The P-value was computed by “wilcox.test” function in R. (**B**) The boxplot demonstrating the editing level among subtypes in TCGA dataset. The P-value was computed by “aov” function in R. (**C**) The Kaplan–Meier overall survival curves of patients were grouped by the editing level in TCGA database. The high and low editing levels were determined by the median value of editing level.

The above results showed that the nonsynonymous RNA editing was enriched in the ubiquitination sites in various cancer types. However, how these nonsynonymous RNA editing sites impacted the tumorigenesis remain uncharacterized. To obtain a comprehensive view of nonsynonymous RNA editing level on ubiquitination site in cancer, we firstly focused on the difference between tumor and para-tumor samples in twelve cancer types. For each cancer types, we identified the nonsynonymous RNA editing on ubiquitination site with significant editing level between tumor and para-tumor samples. Most of the editing level of these nonsynonymous RNA editing were not significant different in cancer. However, the results still showed that the RNA editing level of three sites was significantly different between tumor and para-tumor samples in various samples (Suppl. Fig. S6A). For example, the editing level on two ubiquitination sites, chr1:110256304 on GSTM5 and chr4:10080600 on WDR1, were significantly different between tumor and para-tumor samples in Bladder Urothelial Carcinoma (BLCA) and Head and Neck squamous cell carcinoma (HNSC), respectively (Fig. 5A). Furthermore, we noted that these two sites displayed opposite pattern between tumor and para-tumor samples. One editing site, chr4:10080600 on WDR1, was under-editing in tumor samples. While another site, chr1:110256304 on GSTM5, was over-editing in tumor samples (Fig. 5A). The opposite pattern of editing level may suggest the diverse function of nonsynonymous RNA editing on ubiquitination site in tumorigenesis, reflecting a complex regulation mechanism of nonsynonymous RNA editing on ubiquitination site.

Then we checked the distribution of RNA editing on ubiquitination site among different subtypes in each cancer. We identified five editing sites displaying significantly different editing level among subtypes (Suppl. Fig. S6B). Again, the RNA editing level of two sites, chr1:110256304 on GSTM5 and chr4:10080600 on WDR1, on ubiquitination site was significantly different among different subtypes in two cancer types (Fig. 5B). We also identified other RNA editing sites on ubiquitination site which were significantly distributed among different subtypes including chrX:153062943 on SSR4, chr7:100887329 on FIS1 and chr17:26902513 on ALDOC (Suppl. Fig. S6B).

Finally, we checked the correlation between clinical outcome and the editing level on the ubiquitination sites. To our surprising, chr1:110256304 on GSTM5, chr19: 40485814 on PSMC4 and chrX:153062943 on SSR4 were correlated with overall survival ratio (OS) and identified as prognostic editing sites in Glioblastoma multiforme (GBM), Bladder Urothelial Carcinoma (BLCA) and Kidney renal clear cell carcinoma (KIRC), respectively (Fig. 5C). Besides, we also identified that the editing site, chr1:110256304 on GSTM5, was also correlated with disease free survival (DFI) and progression free survival (PFI) in BLCA (Suppl. Fig. S6C).

In summary, the RNA editing on ubiquitination site posed an impact on clinical features and correlated with clinical outcome, especially for the sites on GSTM5, WDR1, SSR4 and PSMC4. It strongly indicated that RNA editing on ubiquitination site may play a vital role in tumorigenesis and correlated with clinical outcome. Together, above observations could help us understand the role of nonsynonymous RNA editing on ubiquitination site involved in the tumorigenesis.

### The function of genes harboring RNA editing on ubiquitination site were specifically enriched in the immune response

**Figure 6:**
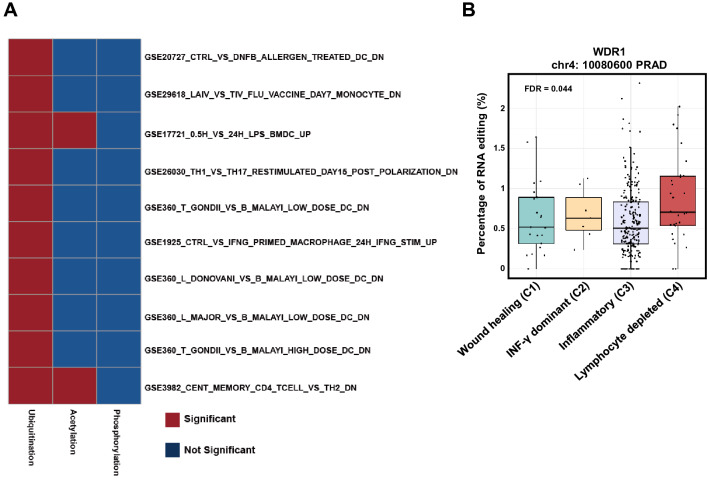
The enrichment analysis of nonsynonymous RNA editing on ubiquitination site. (**A**) The heatmap showing the enrichment of genes harboring nonsynonymous RNA editing in immune response pathway. (**B**) The boxplot demonstrating the editing level among immune subtypes in TCGA dataset. The P-value was computed by “aov” function in R.

Next interesting question was how these genes harboring RNA editing on ubiquitination site function in cell and have a significant impact on the tumorigenesis. To answer this question, we explored the possible function of these genes. The GSEA enrichment analysis revealed that these genes harboring RNA editing sites on ubiquitination site were enriched in the immune response against all genes harboring ubiquitination sites as background (Fig. 6A; Suppl. Fig. S7A). We used all genes harboring ubiquitination sites as background to test whether genes harboring edited ubiquitination sites were specifically enriched over the background. To further demonstrate the specificity of enrichment, we also analyzed the enrichment of genes harboring RNA editing sites on acetylation and phosphorylation site. The results showed that the significant enrichments were not witnessed on phosphorylation and limited on acetylation probably due to the overlapping sites between acetylation and ubiquitination (Figs. 2A, 6A).

To confirm the enrichment analysis, we also investigated the distribution of RNA editing on ubiquitination site among different immune subtypes in TCGA dataset. Three of these sites, chr7:100887329 on FIS1 in HNSC, chrX:153062943 on SSR4 in LUSC and chr4:10080600 on WDR1 in PRAD, were significant different among immune subtypes (FDR < 0.1) (Fig. 6B; Suppl. Fig. S7B).

## Discussion

In this study, we explored the potential function of nonsynonymous RNA editing and found that it was significantly overlapped with ubiquitination site. Since the AA altered by nonsynonymous RNA editing was not the target for ubiquitination, it indicated that the nonsynonymous RNA editing could control the level of ubiquitination. The most well studied role of ubiquitination is targeting proteins for degradation by the proteasome^28^. Besides, ubiquitination also carried out several important functions in DNA repair and the activation of protein kinases^29^. Furthermore, the previous studies showed that the ubiquitination regulate multiple signaling pathways in cell, especially in cancer^30–32^.

Since almost of all ubiquitination events occurred on K not on E or R (Suppl. Fig. S1A), it is straight forward that the protein translated from edited mRNA could not be affected by ubiquitination and escaped from the regulation by ubiquitination. Nonsynonymous RNA editing could decrease the level of ubiquitination and protected protein from degradation or altered the signaling pathways in cell. As far as we known, this is the first work to link the nonsynonymous RNA editing and ubiquitination.

Although we just identified no more than twenty nonsynonymous RNA editing from TCGA dataset located on ubiquitination site, It probably due to the sequencing depth. The previous study showed that the number of identified editing sites was significantly correlated with the mappable bases in present sequencing depth and didn`t reach the platform stage^13^. It meant that more and more nonsynonymous RNA editings on ubiquitination site will be identified along the increasing of sequencing depth. Furthermore, analysis for enrichment of nonsynonymous editing sites unique to the tumor sample could be the best way to explore the role of editings on ubiquitination during tumorigenesis. However, it impossible to perform such analysis due to the limited number of editing sites unique to cancer made and waited for the increasing of sequencing depth.

Although, the previous study showed that RNA editing is involved in the immune response^33–35^ and the ubiquitination is also linked to the immune response^36,37^. Our analysis revealed that genes harboring nonsynonymous RNA editing on ubiquitination site specifically enriched in the immune response pathway. It strongly indicated that nonsynonymous RNA editing may regulate the immune response mediated by controlling the level of ubiquitination during tumorigenesis. Our study shed a light on a novel regulation mechanism of nonsynonymous RNA editing.

Finally, we witnessed that some editings on ubiquitination site were significantly differential edited between tumor and para-tumor samples and among subtypes and correlated with clinical outcome. However, the editing level of these prognostic editings was quite low. It was still significantly correlated with clinical features. Furthermore, the enrichment test was also significant using total genes harboring ubiquitination sites as background. We considered these evidence was convincing, although it may not be fully solid because of low editing level. Besides, the previous study showed that the sequencing depth for RNA editing discovery was far more than enough, since the number of informative editing sites were tightly correlated with sequencing depth^13^. The actual editing level of these editings could be under-estimated due to the sequencing depth and sampling error.

## Methods

### Data download

All methods were carried out in accordance with relevant guidelines and regulations.

The protein modification data including acetylation, methylation, O-GalNAc, O-GlcNAc, phosphorylation, sumoylation and ubiquitination were downloaded from PhosphoSitePlus^27^ (https://www.phosphosite.org). The A-to-I RNA editing sites for human were downloaded from REDIportal (http://srv00.recas.ba.infn.it/atlas/download.html) and DARNED database^11,12^ (https://darned.ucc.ie/). The A-to-I RNA editing sites in Gabay2022 were downloaded from the supplementary data in their paper^8^. The RNA editing data for TCGA dataset was downloaded from Synapse under the accession number syn2374375^13^https://www.synapse.org/#!Synapse:syn2374375 (). The coordinates of A-to-I RNA editing sites analyzed in our work were based on hg19 human genome. The coordinates of A-to-I RNA editing sites in Gabay2022 were converted into hg19 human genome by liftover. The proteomic data for breast and ovarian cancer was downloaded from the National Cancer Institute’s Clinical Proteomic Tumor Analysis Consortium (CPTAC) project^38,39^ (https://cptac-data-portal.georgetown.edu/). The somatic mutation data to check mismatched sites on peptides was downloaded from UCSC Xena (https://xenabrowser.net/datapages/).

### Conversion of the position of modification sites from protein level to genome level

The position of modification sites on protein level were extracted from the data downloaded from PhosphoSitePlus. The genomic range of corresponding amino acids for each modification site were mapped onto the hg19 human genome by “biomaRt” package in Bioconductor^40^. We analyzed the amino acid changes by the codon sequence before and after editing. Then the genomic position of modification site was overlapped with the position of nonsynonymous RNA editing site in REDIportal, Gabay2022 and DARNED database as well as in seventeen cancer types in TCGA dataset. We also counted and summarized the percentage of modified AA in PhosphoSitePlus database as the possibility of specific AA to be modified for each modification type.

We applied one-sided Fisher’s exact test in R to test the significance between two percentages, which was commonly used to test the significant overlaps in genomic study^41^. Taking the overlaps between (non)synonymous RNA editings and ubiquitination sites in DARNED database and ubiquitination sites in PhosphoSitePlus database as an example, we tabulated the counts of (non)synonymous RNA editings overlapped and non-overlapped with ubiquitination site. The resulting table is 2X2 matrix and contains four digitals which are the counts of nonsynonymous RNA editings overlapped with ubiquitination site, the counts of nonsynonymous RNA editings nonoverlapped with ubiquitination site, the counts of synonymous RNA editings overlapped with ubiquitination site and the counts of synonymous RNA editings nonoverlapped with ubiquitination site. The one-side Fisher’s exact test was performed to test the significance between the distributions of (non)synonymous RNA editings overlapped with ubiquitination site.

### Proteomics data analysis

The BRCA and OV MS datasets were downloaded from The National Cancer Institute’s Clinical Proteomic Tumor Analysis Consortium (CPTAC) in mzML-format. The data processing was basically followed the previous works^13,20^. The MS data was converted by “MSConvert” in ProteoWizard with unchecking the “Use zlib compression” option.

Then we used “sapFinder” in Bioconductor for the database construction, peptide matching and identification of peptides comprising mismatching AA due to RNA editing^42^. We separately constructed two mismatch searching lists for identification of the nonsynonymous RNA editing events in BRCA and OV based on GENCODE gene annotation which was downloaded from UCSC Table browser (GENCODE.V37lift37). The coordinates of gene annotation were based on hg19 human genome. The searching lists for BRCA were derived from BRCA dataset and used for searching peptides comprising mismatching AA in BRCA MS data, respectively. Since there was no RNA editing data for OV, we merged all RNA editing events in available seventeen TCGA datasets and constructed the mismatch searching list for OV based on the merged list.

For mismatch searching, we added two more fixed modifications, iTRAQ 4-plex of N-terminal and lysine (144.10 Da) and oxidation on methionine (15.99 Da) and two variable modifications, acetylation of protein N-term (42.01 Da) and deamination of asparagine (0.98 Da). The other options in “sapFinder” were kept as default parameters. We read the final output from “peptideSummary.txt” file and picked the peptides comprising mismatching AA due to RNA editing. Since the limited number of edited peptides were identified. We just manually checked the information of somatic mutations in VCF format files for corresponding patient to make sure that the mismatched AA in these peptides was not due to the somatic mutation.

### Identification of RNA editing on the ubiquitination sites related to the cancer progress in TCGA

The information of subtype for each patient in TCGA dataset was download from previous study^43,44^. The significance was computed by “wilcox.test” function in R. The raw P-value was adjusted by “p.adjust” function with “method = fdr” in R for each cancer type.

Since there are lots of editing sites which editing level are not available due to sequencing depth in some samples, we only kept the sites available in at least three samples in any groups (e.g., tumor group, para-tumor group and subtype groups) when identifying the differential editing sites.

### Survival analysis in TCGA datasets

The survival analysis was basically performed as previously studies^45,46^. For each cancer dataset, survival information was retrieved from the “TCGA-CDR” dataset^47^. Since the information of disease-specific survival is approximated in most of TCGA datasets, we excluded it from our analysis and kept other three endpoints (overall survival (OS), disease-free interval (DFI), and progression-free interval (PFI)).

Cox models were run with the “coxph” function from the “survival” package in R. For survival plots, we divided the samples into two groups based on the editing level. The cutoff to classify the groups was the median value of editing level. Then Cox models were run with the “coxph” function, and the equation is “coxph(Surv(time,censor) ∼ group).” We picked the editing sites with Logrank p-value ≤ 0.05 and considered that these editing sites as the sites in which the editing level correlated with different survival outcome.

### Gene set enrichment analysis (GSEA)

Gene Set Enrichment Analysis (GSEA) was performed using genes harboring nonsynonymous RNA editing on specific modification in “clusterProfiler” in R^48^. The background gene set for enrichment analysis was the genes with corresponding modification annotated in PhosphoSitePlus database. The gene sets for enrichment analysis were immunologic signature gene sets (C7) from the Molecular Signatures Database (MSigDB)^49^ (http://www.gsea-msigdb.org/gsea/msigdb/annotate.jsp).

## Supplementary Information

Supplementary Information 1.

Supplementary Information 2.

Supplementary Information 3.

Supplementary Information 4.

Supplementary Information 5.

Supplementary Information 6.

Supplementary Information 7.

Supplementary Information 8.

Supplementary Information 9.

Supplementary Information 10.

Supplementary Information 11.

Supplementary Information 12.

## Data Availability

The raw measurements are provided in the Supplemental figures and files.
