# Supplementary material for: A-to-I nonsynonymous RNA editing was significantly enriched in the ubiquitination site and correlated with clinical features and immune response
Source: Sci Rep. 2022 Sep 5;12:15079. doi: 10.1038/s41598-022-18926-x (PMC9445000; doi:10.1038/s41598-022-18926-x)
Supplement: Supplementary file 1 — Supplementary Information 1. [file 41598_2022_18926_MOESM1_ESM.pdf]

Figure S1

A

Modification type	Modified AA	Counts of modified AA	Percentage of modified AA
Acetylation	K	22810	99.93%
Acetylation	S	10	0.04%
Acetylation	T	5	0.02%
Methylation	R	11148	68.10%
Methylation	K	5210	31.83%
Methylation	C	8	0.05%
Methylation	H	3	0.02%
Methylation	L	1	0.01%
O-GalNAc	T	1265	60.18%
O-GalNAc	S	827	39.34%
O-GalNAc	Y	10	0.48%
O-GlcNAc	S	256	58.72%
O-GlcNAc	T	180	41.28%
Phosphorylation	S	141321	59.10%
Phosphorylation	T	58549	24.49%
Phosphorylation	Y	39223	16.40%
Phosphorylation	H	14	0.01%
Phosphorylation	R	2	0.00%
Phosphorylation	K	2	0.00%
Phosphorylation	D	1	0.00%
Phosphorylation	G	1	0.00%
Phosphorylation	V	1	0.00%
Phosphorylation	P	1	0.00%
Sumoylation	K	8411	100.00%
Ubiquitination	K	97760	100.00%
Ubiquitination	C	2	0.00%
Ubiquitination	S	1	0.00%
Ubiquitination	R	1	0.00%
