# Supplementary figures and images for: A-to-I nonsynonymous RNA editing was significantly enriched in the ubiquitination site and correlated with clinical features and immune response
Source: Sci Rep. 2022 Sep 5;12:15079. doi: 10.1038/s41598-022-18926-x (PMC9445000; doi:10.1038/s41598-022-18926-x)

Figure S2

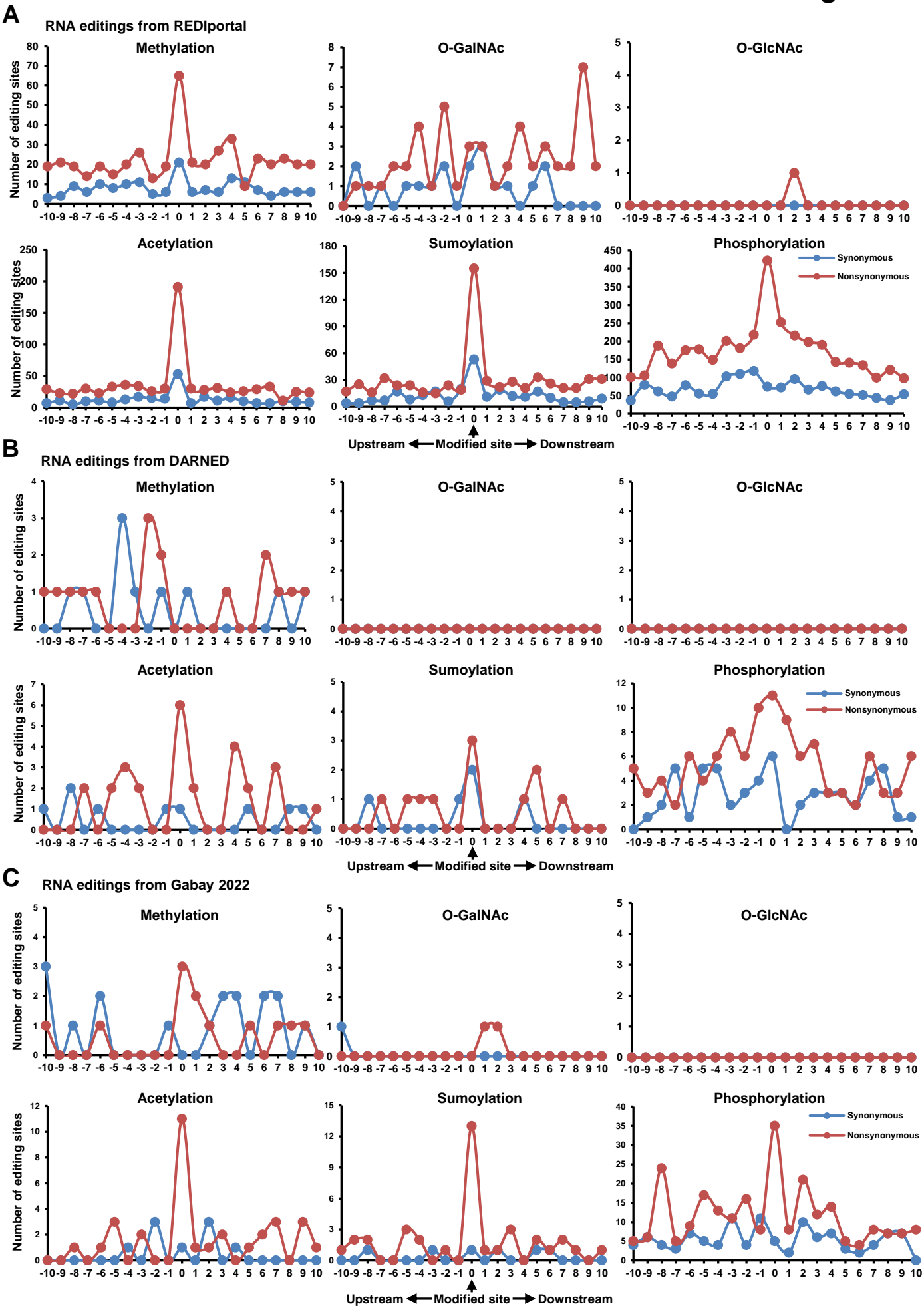

Supplement: Supplementary file 2 — Supplementary Information 2. [file 41598_2022_18926_MOESM2_ESM.pdf]

Figure S3

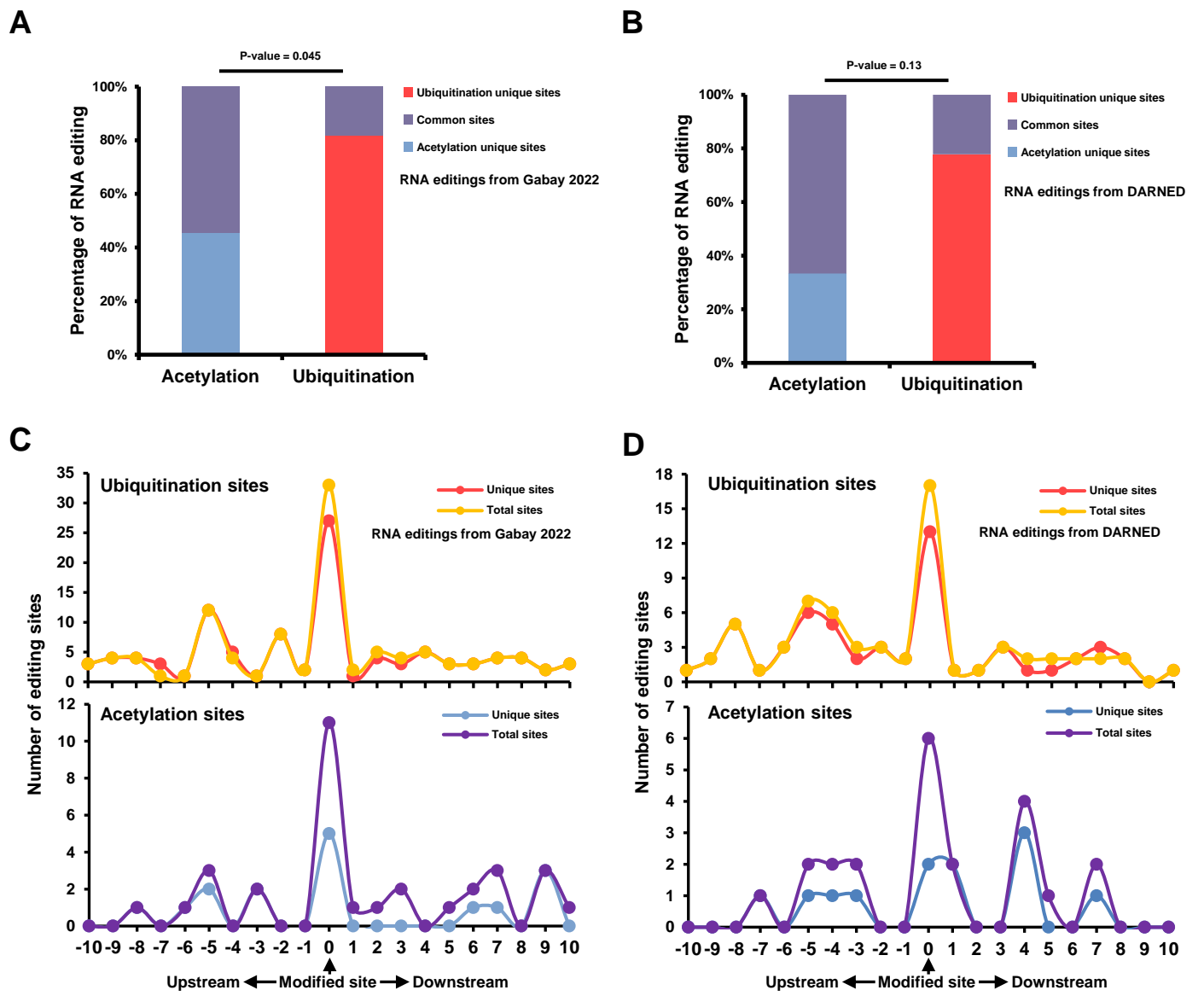

Supplement: Supplementary file 3 — Supplementary Information 3. [file 41598_2022_18926_MOESM3_ESM.pdf]

Figure S4

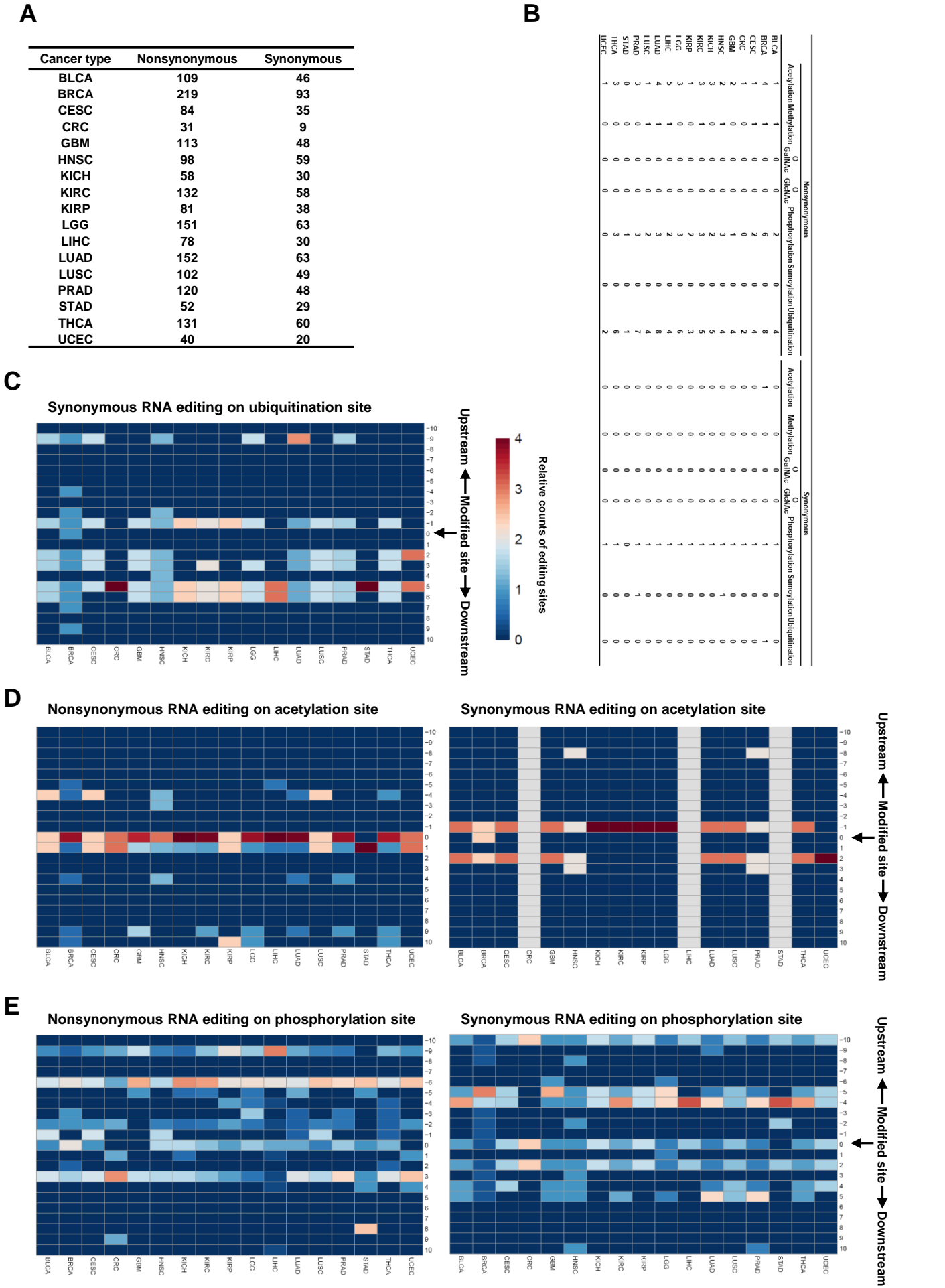

Supplement: Supplementary file 4 — Supplementary Information 4. [file 41598_2022_18926_MOESM4_ESM.pdf]
