# Supplementary material for: A-to-I nonsynonymous RNA editing was significantly enriched in the ubiquitination site and correlated with clinical features and immune response
Source: Sci Rep. 2022 Sep 5;12:15079. doi: 10.1038/s41598-022-18926-x (PMC9445000; doi:10.1038/s41598-022-18926-x)
Supplement: Supplementary file 5 — Supplementary Information 5. [file 41598_2022_18926_MOESM5_ESM.pdf]

Figure S5

A

Gene	Cancer type	evalue	charge	mass	mz	delta_da	delta_ppm	rt	FDR	Qvalue	Peptide
GSTM5	BRCA	0.034	3	2003.942	2004.972	0.016	7.984261	2467.317	0	0	YIARKHNLCGETEEER
PSMC4	BRCA	0.057	3	2368.245	2369.261	0.0026	1.097859	5001.864	0	0	LARENAPAIIFIDEIDAIATK
PSMC4	BRCA	0.066	3	2368.245	2369.252	-0.0068	-2.87132	4091.34	0	0	LARENAPAIIFIDEIDAIATK
PSMC4	BRCA	0.08	3	2368.245	2369.267	0.0082	3.462479	4501.924	0	0	LARENAPAIIFIDEIDAIATK
USP4	OV	0.099	3	1890.928	1891.944	0.0031	1.639407	2352.321	0	0	PDAVVAEEAWENHRLR
PSMC4	OV	0.027	2	1261.702	1262.718	0.003	2.377741	1284.522	0	0	MVRDVFRLAR
ALDOC	OV	0.027	2	1396.667	1397.689	0.0082	5.871118	914.932	0	0	PHSYPALSAEQR
GSTM5	OV	0.066	3	2003.942	2004.95	-0.0051	-2.54498	1943.738	0	0	YIARKHNLCGETEEER
GSTM5	OV	0.054	3	2003.942	2004.957	0.0015	0.748525	2126.489	0	0	YIARKHNLCGETEEER
