# Supplementary material for: A-to-I nonsynonymous RNA editing was significantly enriched in the ubiquitination site and correlated with clinical features and immune response
Source: Sci Rep. 2022 Sep 5;12:15079. doi: 10.1038/s41598-022-18926-x (PMC9445000; doi:10.1038/s41598-022-18926-x)
Supplement: Supplementary file 6 — Supplementary Information 6. [file 41598_2022_18926_MOESM6_ESM.pdf]

Figure S6

A

Cancer type	Editing site	Median of editing level in tumor	Median of editing level in para-tumor	Sample number in tumor	Sample number in para-tumor	P-value	FDR
BLCA	chr1|110256304|GSTM5|+	0	0.03125	99	19	3.28E-06	1.31E-05
THCA	chr1|110256304|GSTM5|+	0	0.038462	160	54	3.89E-05	0.000234
HNSC	chr4|10080600|WDR1|-	0.005083	0.003035	426	42	0.000101	0.000405
PRAD	chr17|80041164|FASN|-	0	0	374	52	0.00426	0.029823
PRAD	chr4|10080600|WDR1|-	0.005769	0.00469	374	52	0.013356	0.046747

B

Cancer type	Editing site	P-value	FDR
PRAD	chrX|153062943|SSR4|+	0.000135	0.000942
UCEC	chr4|10080600|WDR1|-	0.01444	0.02888
LIHC	chr7|100887329|FIS1|-	0.012055	0.04822
GBM	chr4|10080600|WDR1|-	0.011979	0.047915
HNSC	chr1|110256304|GSTM5|+	0.004749	0.018994
LUSC	chr4|10080600|WDR1|-	0.008773	0.017547
LUSC	chr1|110256304|GSTM5|+	0.008691	0.017547
LGG	chr17|26902513|ALDOC|-	1.72E-05	0.000103

C

Editing site	Gene	Cancer type	Median for high editing (%)	Median for low editing (%)	Logrank P-value	Survival type
chr1|110256304	GSTM5	GBM	8.33	2.51	0.006876	OS
chr19|40485814	PSMC4	BLCA	0.19	0	0.010779	OS
chrX|153062943	SSR4	KIRC	0.06	0	0.017387	OS
chrX|153062943	SSR4	KICH	0.07	0	0.026162	OS
chr1|110256304	GSTM5	BLCA	3.70	0	0.016454	DFI
chr4|10080600	WDR1	LGG	1.15	0.37	0.035648	DFI
chr1|110256304	GSTM5	BLCA	3.70	0	0.001768	PFI
