# Supplementary material for: A-to-I nonsynonymous RNA editing was significantly enriched in the ubiquitination site and correlated with clinical features and immune response
Source: Sci Rep. 2022 Sep 5;12:15079. doi: 10.1038/s41598-022-18926-x (PMC9445000; doi:10.1038/s41598-022-18926-x)
Supplement: Supplementary file 7 — Supplementary Information 7. [file 41598_2022_18926_MOESM7_ESM.pdf]

Figure S7

A

Gene Set Name	GeneRatio	BgRatio	p-value	FDR
GSE20727_CTRL_VS_DNFB_ALLERGEN_TREATED_DC_DN	3/10	111/10409	1.34E-04	2.72E-02
GSE360_T_GONDII_VS_B_MALAYI_LOW_DOSE_DC_DN	3/10	125/10409	1.91E-04	2.72E-02
GSE29618_LAIV_VS_TIV_FLU_VACCINE_DAY7_MONOCYTE_DN	3/10	129/10409	2.09E-04	2.72E-02
GSE17721_0.5H_VS_24H_LPS_BMDC_UP	3/10	130/10409	2.14E-04	2.72E-02
GSE1925_CTRL_VS_IFNG_PRIMED_MACROPHAGE_24H_IFNG_STIM_UP	3/10	138/10409	2.56E-04	2.72E-02
GSE360_L_MAJOR_VS_B_MALAYI_LOW_DOSE_DC_DN	3/10	142/10409	2.78E-04	2.72E-02
GSE26030_TH1_VS_TH17_RESTIMULATED_DAY15_POST_POLARIZATION_DN	3/10	143/10409	2.84E-04	2.72E-02
GSE360_T_GONDII_VS_B_MALAYI_HIGH_DOSE_DC_DN	3/10	144/10409	2.90E-04	2.72E-02
GSE360_L_DONOVANI_VS_B_MALAYI_LOW_DOSE_DC_DN	3/10	147/10409	3.08E-04	2.72E-02
GSE3982_CENT_MEMORY_CD4_TCELL_VS_TH2_DN	3/10	159/10409	3.88E-04	3.09E-02

B

Cancer type	Editing site	P-value	FDR
HNSC	chr7#100887329|FIS1|-	0.002840717	0.011362867
LUSC	chrX#153062943|SSR4|+	0.024196769	0.072590308
PRAD	chr4#10080600|WDR1|-	6.30E-03	0.044104671
