# Supplementary material for: A-to-I nonsynonymous RNA editing was significantly enriched in the ubiquitination site and correlated with clinical features and immune response
Source: Sci Rep. 2022 Sep 5;12:15079. doi: 10.1038/s41598-022-18926-x (PMC9445000; doi:10.1038/s41598-022-18926-x)
Supplement: Supplementary file 12 — Supplementary Information 12. [file 41598_2022_18926_MOESM12_ESM.docx]

## **Figure S1. The counts of amino acids (AAs) affected by nonsynonymous RNA editing.**

**(A).** Table summarizing the counts of amino acids (AAs) affected by nonsynonymous RNA editing in three RNA editing databases.

## **Figure S2. The distribution of RNA editing**

**(A-C).** The distribution of synonymous (blue) and nonsynonymous (red) RNA editing from REDIportal (A), DARNED (B) and Gabay2022 (C) databases on various modification site and flanking regions.

## **Figure S3. The distribution of nonsynonymous RNA editings overlapped with ubiquitination sites**

**(A-B).** Bar plot demonstrating the percentage of nonsynonymous RNA editing in Gabay2022 (A) and DARNED (B) databases located in the ubiquitination unique and acetylation unique sites. The P-value was computed by one-sided “fisher.test” function in R. **(C-D).** The distribution of nonsynonymous RNA editing from Gabay2022 (C) and DARNED (D) databases on total and ubiquitination unique site and flanking regions (upper) and on total and acetylation unique site and flanking regions (bottom).

## **Figure S4. The distribution of RNA editing in TCGA dataset**

**(A).** Table summarizing the counts of nonsynonymous and synonymous RNA editing in each cancer type. **(B).** Table summarizing the counts of nonsynonymous and synonymous RNA editing overlapped with modification sites in each cancer type. **(C).** Heatmap depicting the distribution of synonymous RNA editing on ubiquitination site and flanking region for each cancer type. **(D).** Heatmap depicting the distribution of nonsynonymous and synonymous RNA editing on acetylation site and flanking region for each cancer type. **(E).** Heatmap depicting the distribution of nonsynonymous and synonymous RNA editing on phosphorylation site and flanking region for each cancer type.

## **Figure S5. Peptides** **harboring edited ubiquitination site were identified from CPTAC dataset**

**(A).** Table summarizing the peptides harboring edited ubiquitination site identified from CPTAC dataset.

## **Figure S6. The distribution of** **nonsynonymous RNA editing on ubiquitination site in TCGA database**

**(A).** Table summarizing the nonsynonymous RNA editing sites which were differential edited between tumor and para-tumor samples. **(B).** Table summarizing the nonsynonymous RNA editing sites which were differential edited among subtypes for each cancer type. **(C).** Table summarizing the nonsynonymous RNA editing sites which were significantly correlated with clinical outcome.

## **Figure S7. The enrichment analysis for the genes** **harboring edited ubiquitination site**

**(A).** Table summarizing the significantly enrichment pathway for the genes harboring edited ubiquitination site. **(B).** Table summarizing the nonsynonymous RNA editing sites which were differential edited among immune subtypes for each cancer type.

## **Table S1. AAs affected by nonsynonymous RNA editing in REDIportal database**

## **Table S2. AAs affected by nonsynonymous RNA editing in DARNED database**

## **Table S3. AAs affected by nonsynonymous RNA editing in Gabay2022 database**

## **Table S4. Nonsynonymous and synonymous RNA editing sites identified from 17 cancer types in TCGA datasets**
